# Supplementary material for: Deep-sea mining discharge can disrupt midwater food webs
Source: Nat Commun. 2025 Nov 6;16:9575. doi: 10.1038/s41467-025-65411-w (PMC12592452; doi:10.1038/s41467-025-65411-w)
Supplement: Supplementary file 1 — Supplementary Infomation [file 41467_2025_65411_MOESM1_ESM.pdf]

Supporting Information for

Deep-sea mining discharge can disrupt midwater food webs

Michael H. Dowd [1,*], Victoria E. Assad [1,†], Alexus E. Cazares-Nuesser [1,†], Jeffrey C. Drazen [1], Erica Goetze [1], Angelicque E. White [1], Brian N. Popp [2]

1: Department of Oceanography, University of Hawai'i at Mānoa, Honolulu, HI, 96822

2: Department of Earth Sciences, University of Hawai'i at Mānoa, Honolulu, HI, 96822

* Corresponding author; email: mdowd3@hawaii.edu

† These authors contributed equally: Victoria E. Assad, Alexus E. Cazares-Nuesser

Supporting Information Text

Supplementary Note 1: Dissolved Oxygen in Plume

We expected the dissolved oxygen content in the plume to be higher than that of seawater at the discharge depth. Dissolved oxygen concentration near the seafloor (~4250-4310 m) measured using calibrated sensors on the CTD package during the two hydrographic casts was ~158 ± 3 μM (n=2, TC_013, HC_051). We determined the dissolved oxygen concentration in water samples collected from the riser pipe aboard the *Hidden Gem* prior to release into the midwater during mining operations using a potentiometric end-point titration procedure (Winkler Titration). The equipment and protocols of the Hawaii Ocean Time-series program were followed (see Hawaii Ocean Time-series protocols). Dissolved oxygen concentrations in the riser water were 994 ± 188 μM (Table S8), far exceeding those from near the abyssal seafloor or from surface waters, likely due to the use of compressed air injected at ~2000m to lift nodules up the riser pipe from the seafloor.

Within a plume, beam transmission ranged from 28.09-89.99% over depth interval 1253-1282m, under normal mining operations (MC_004), we found an average dissolved oxygen concentration of 60.0 ± 0.5 μM. This sampling represented plume conditions within 300-1000m of the point of discharge. We calculated that the time spent within the described plume conditions represents 38.4% of the casts, based on total number of scans by the CTD of the cast versus the number of scans within the 28.09-89.99% beam transmission range. In contrast, during three background CTD casts in this region, when no plume was being generated and beam transmission exceeded 90%, dissolved oxygen concentrations at 1250m was 59.8 ± 0.6 μM (n=3, HC_050,

HC_051, HC_052). Although the dissolved oxygen concentration within the plume was only slightly higher than background levels, the difference was not significant. However, a significant negative linear correlation ($P < 0.0001$) was observed between beam transmission and dissolved oxygen concentrations within the plume (Fig. S3). These observations indicate that the dissolved oxygen concentration of the discharge seawater is higher than background dissolved oxygen concentrations at the depth of discharge, likely ranging from about 160 µM to as high as ~1 mM.

While the volume of oxygenated water injected at the proposed discharge depth of ~1250m is unlikely to significantly impact oxygen concentrations at that depth, the effect could be more pronounced if the discharge occurs within the oxygen minimum zone (OMZ) core (dissolved oxygen <1 µM) or at oxyclines (dissolved oxygen <15 µM). Organisms adapted to near anoxic conditions (<10 µM) are known to be sensitive to even small changes in oxygen concentration, often within a few micromoles [1]. This suggests that while the plume may not significantly alter oxygen concentrations at ~1250m, if the discharge occurs within the OMZ core or near the oxyclines, the resulting changes could alter the distribution of organisms.

Supplementary Note 2: CSIA-AA

Compound specific isotope analysis of amino acids (CSIA-AA) is a technique that allows the differentiation of different food sources and evaluation of trophic position in organisms [2,3]. Nitrogen isotopic analysis of amino acids shows predictable, non-uniform isotopic fractionation among different amino acids (AAs) [4]. “Trophic” AA $\delta^{15}N$ values (Tr; aspartic acid [Asp], glutamic acid [Glu], alanine [Ala], isoleucine [Ile], leucine [Leu], valine [Val], proline [Pro]) become predictably and significantly higher with each trophic transfer [5] while “Source” AA $\delta^{15}N$ values (Src; glycine [Gly], serine [Ser], methionine [Met], phenylalanine [Phe], lysine [Lys]) show minimal change during a trophic transfer [5]. As such, source amino acid $\delta^{15}N$ values can be used to identify different food sources at the base of the food web [6]. We used the source amino acids Phe and Lys because we could reliably measure their $\delta^{15}N$ values in particles and metazoans.

The $\delta^{13}C$ values of essential amino acids (EAA; Ile, Leu, Lys, Phe, Thr, Val, Met), which cannot be synthesized by metazoans [7], similarly remain unchanged during trophic transfer [8]. These EAA can also produce a unique “fingerprint” of $\delta^{13}C$ values depending on biosynthetic origin in the original primary producer [9,10]. Like source amino acid $\delta^{15}N$ values, $\delta^{13}C$ values of essential amino acids can be used to differentiate food sources making up the base of food web. We used the essential amino acid Leu because $\delta^{13}C$ values were significantly different between particle size fractions it was reliably measured in particles and metazoans. Combined, $\delta^{15}N_{Src}$ and $\delta^{13}C_{EAA}$ can be used to differentiate food sources at the base of the food web and identify the diet of metazoans using mixing models.

Supplementary Note 3: Zooplankton Community Analysis

DNA Extraction, Amplification, and Sequencing

Zooplankton DNA was extracted from 9 MOCNESS tows in spring (5 tows, 108 samples) and fall (4 tows, 72 samples) at the collector test site. Two size fractions (0.2–1.0 mm, >5.0 mm) for each net were processed using a modified OMEGA E.Z.N.A. Blood Maxi kit protocol. A ~365-bp fragment of the V1-V2 region of nuclear 18S rRNA was amplified using primers F04 [5'- GCTTGTCTCAAAGATTAAGCC -3'] and R22mod [5'- CCTGCTGCCTTCCTTRGA-3'] [11,12]. The full primer sequence including Illumina overhang adapters was as follows: F04 [5'-TCGTCGGCAGCGTCAGATGTGTATAAGAGACAGGCTTGTCTCAAAGATTAAGCC-3'] and R22mod [5'-GTCTCGTGGGCTCGGAGATGTGTATAAGAGACAGCCTGCTGCCTTCCTTRGA-3']. Primers were synthesized by Integrated DNA Technologies (25 nmole DNA oligos). PCR amplification was performed in duplicate for each sample, and products were pooled and purified with Agencourt® AMPure® XP beads (Beckman Coulter®). Purified products were normalized to 2ng/uL, indexed using the NexteraTM DNA 180 library Prep Kit (Illumina), and sequenced on MiSeq Illumina runs using V3 chemistry and paired-end sequencing (2×300 bp). Extraction and PCR negative controls (N=19) were included in PCR amplification and sequencing to detect contamination (Supplementary Data 3).

Bioinformatics

Demultiplexed reads were denoised using DADA2 within Qiime2, with truncation at 270 bp (forward) and 235 bp (reverse) followed by paired-end read merging [13,14] and chimera detection via the denovo consensus approach. Rarefaction confirmed sufficient sequencing depth. 18S reads were classified using a Naïve-Bayes Classifier in Qiime2 [15]

with the SILVA 18S rRNA (v132, 99% similarity; [16,17]), Pr2 (v5.0.0; [16]), and MetaZooGene 18S (v2024-m02-17; [18,19]) databases. Sequences were also compared to GenBank [20] using Blastn and Megablast, with a 99% similarity threshold applied. Taxonomic assignments were determined by identifying the last common ancestor (LCA) among all outputs using the "biohelper" R package. Contaminants were removed with the "decontam" R package [21] using both frequency and prevalence criteria.

Functional Ecology Analysis

Zooplankton collections made between 700–1500 m were the focus of this analysis, as this region of the water column is below the OMZ and encompasses the proposed mining plume. A complete taxon list of unique ASVs and their corresponding taxonomic assignments was generated, including both size fractions, both seasons, and all four MOCNESS nets (700-800 m, 800-1000 m, 1000-1250 m, 1250-1500 m). To focus on ecologically-important or 'core' taxa within this depth horizon, ASVs were filtered to include only those that comprised >0.1% of total relative read abundance within a given tow and that were present in at least two MOCNESS tows (of 9 total). Taxa present at the potential discharge depth (1000-1500 m) are flagged in Supplementary Data 1. Taxa were categorized into trophic functional groups based on literature reports of diet and feeding mode (e.g., [22–24], Supplementary Data 1). Categorizations were made considering observations reported for the same species in other regions of the global ocean, congeneric species expected to have similar feeding mode, or for feeding specialization at the Family level in some cases (e.g., Aetideidae, Diaxidae, Phaennidae, Scolecithricidae, Tharybdidae are detritivores with specialized sensory setae that aid in detection of detrital food particles in the water column (e.g., [25]). Fish were excluded from our calculations to focus on the mesozooplankton assemblage.

Relative read abundance is reported across taxonomic groups, which often correlates with biomass for dominant taxa (e.g., [26,27]).

Zooplankton Functional Group Results

Below 700 m, a diverse zooplankton assemblage included copepods, hydrozoan and scyphozoan cnidarians, decapods, euphausiids, chaetognaths, tunicates, polychaetes, ostracods, and gastropods, among others. 186 unique zooplankton ASVs, corresponding to 79 distinct taxa, made up the core community at these depths (Supplementary Data 1; curated taxon list). The small size fraction (0.2-1.0 mm) was dominated by particle-feeding taxa, primarily consisting of calanoid copepods (74-76% of 18S reads). Five families—Metridinidae, Scolecitrichidae, Spinocalanidae, Rostrocalanidae, and Eucalanidae—contributed the highest relative read abundance (44-55% of reads). Other taxa broadly reliant on particles as a food source included ostracods (3-4% of reads; genus *Conchoecia*, family Cypridinidae), oncaeid copepods (2% of reads), and thecosome pteropods (genus *Hyalocylis*; gastropods made up 0.3-1% of reads, including predatory pneumodermatid gymnosomes). Euphausiids represented 4-6% of reads in the small size fraction and are likely partially reliant on detrital material at depth (see below). Other copepods (3-4% of reads) included harpacticoids in the Family Aegistidae that have no apparent dietary reports in the literature.

In the large zooplankton size fraction (>1.0 mm), calanoid copepods remained dominant (57-58% of reads), but the relative abundance of gelatinous taxa increased with depth. Siphonophores (Apolemiidae, Erennidae) accounted for 17-26% of reads below 700 m, compared to 0.3-4% of reads in shallower waters (0-700 m). Other

gelatinous taxa, including tunicates (Salipidae, Doliolidae; 1-4% of reads), scyphozoans (Paraphyllinidae, Ulmaridae; 0.4-1% of reads), and chaetognaths (*Pseudosagitta*, *Eukrohnia;* 1% of reads), were present at lower relative abundances. These gelatinous organisms may be particularly vulnerable to mining plumes, as suspended particles can adhere to their gelatinous bodies and feeding structures, disrupting buoyancy and leading to increased mucous production and energy requirements (23, 24). Several large crustacean groups also increased in relative abundance with depth. Although midwater shrimp (*Gennadas, Plesionika, Sergia*) and euphausiids (*Bentheuphausia*) are reported to be primarily predatory zooplanktivores, they opportunistically feed on detrital aggregates (e.g., [28–30]). In contrast, oplophorid shrimp are not known to feed on aggregates [31].

Overall, 53% of the core zooplankton taxa below 700 m were classified as particle feeders and 20% as gelatinous zooplankton, both of which may be particularly susceptible to midwater mining plumes. At the potential discharge depth specifically (1000-1500 m), the zooplankton community was composed of 75 distinct taxa, with a slightly higher proportion of particle feeders (55%) and gelatinous taxa (21%).

Supplementary Note 4: Micronekton Functional Group Results

Micronekton collections from 700 – 1500m, including two distinct depth strata, 700 – 1000m and 1000 – 1500m, were the focus of this analysis as they were below the OMZ core and encompassed the proposed mining plume depths. Micronekton were identified to the lowest taxonomic unit (LTU) possible, mostly to species using morphology based taxonomic keys. Trophic functional groups were assigned based on trophic ecology literature or using reports in Fishbase (Supplementary Data 2). For those without species-specific literature, categories were based upon information at the genus or family level expected to have a similar feeding mode. Definitions of trophic groups were based upon those described in Drazen & Sutton 2017 [32]. Note, Euphausiids were found to be an important part of the community captured with the large nets and thus were included amongst the micronekton community. Proportion of total taxa attributed to different functional trophic groups and the proportion of total micronekton density for each trophic group are reported.

The lower oxycline community, from 700 – 1000 m, was composed of 76 taxa with identifiable functional trophic groups (Supplementary Data 2). 59% of these were categorized as zooplanktivores and 38% of the taxa were pelagic micronektonivores (Supplementary Data 2). The community was dominated by the zooplanktivorous bristlemouth fishes *Cyclothone* spp. [32], making up 79.15% of the density in this depth section. Also abundant were the zooplanktivorous bigscale fishes (Family Melamphaidae; [33–35]), making up 6.75% of the density at this depth. The Twospine bigscale, *Scopelogadus bispinosus,* dominated, accounting for ~4% of the 6.75% of the density in the LO. Melamphaids are particularly important at this site as many species, including *Scopelogadus bispinosus* are vertical migrators. Sergestid and Penaid shrimps are also zooplanktivorous [29,36,37] and were common in this depth strata, making up 2.2%

and 1.2% of the density respectively. The pelagic micronektonivores with the highest contribution to total density were the dragonfishes (Family Stomiidae; [32]), specifically those in the genus *Idiacanthus*, making up 1.79% of the density in the LO.

Similar to the LO, the discharge depth (1000-1500m) contained 57 taxa of micronekton with identifiable functional trophic groups (Supplementary Data 2). 61% of species were classified as zooplanktivores. Pelagic micronektonivores represented 35% of the community at this depth. Amongst zooplanktivorous taxa *Cyclothone* spp, comprised 24% of the density at this depth. The most common species in the *Cyclothone* genus, *Cyclothone acclinidens,* represented 7.74% of the total density, Euphausiids also dominated in this depth strata with *Bentheuphausia amblyops* making up 7.72% of the total density, nearly equivalent to *C. acclinidens* and are also zooplanktivorous [38]. The zooplanktivorous lanternfishes (Family Myctophidae; [32]), particularly those in the genus *Lampanyctus* were the most dominant fish besides *Cyclothone* spp., making up 12.81% of the discharge depth density. Myctophids are also important vertical migrators that contribute to the biological carbon pump [39]. The pelagic micronektonivore Caridean shrimp *Acanthephyra brevicarinata*, consumes primarily small fishes in the families Myctophidae and Gonostomatidae, along with Euphausiids and Sergestid shrimps [40] and was 3.97% of the discharge depth community.

Supplementary Note 5: Particle Collection

Size fractionated particulate matter was collected using *in situ* Large Volume Water Transfer Systems (McLane WTS-LV) pumps, equipped with pump heads operating at either 8 L/min or 30 L/min. For the present results, the sampling depths targeted particles at several depths within the mesopelagic zone above the proposed discharge depth (OMZ core ~400 m and lower oxycline ~850 m), and two depths in the upper bathypelagic zone (1,000–1,500 m). Each pump was fitted with tiered 142-mm diameter mini-Multiple Unit Large Volume Filtration System (mini-MULVFS) filter holders [41]. The 30 L/min pumps were equipped with a 53 µm and 6 µm Nylon or polyester screen to collect particles in the size range of 6-53 and >53 µm. The 8 L/min pumps included an additional 0.7 µm glass fiber filter (GFF) to collect particles in the 0.7–6 µm size range. The 30 L/min pumps filtered approximately 5600-6100 L of seawater per collection, while the 8 L/min pumps filtered approximately 190-1350L. Due to particularly low particle concentrations in the mesopelagic and upper bathypelagic zones, several depths were combined, or multiple pumps were deployed at a single depth horizon to ensure sufficient material (~0.5 mg of N) was available for compound-specific isotope analysis of amino acids (CSIA-AA).

GFF filters were subsampled using a MeOH-rinsed 1 cm diameter punch for PC, POC, and PN analysis. The remaining portion of the filter was used for compound specific isotope analysis of amino acids (CSIA-AA). The 6 and 53 µm screens were rinsed with filtered sea water in a clean beaker and split using a Folsom plankton splitter. Proportions were allocated as follows: ¼ to ½ dedicated for PCPN, ¼ dedicated to POC (when analyzed) and the remaining for CSIA-AA. Samples were split immediately after the sampling cast at sea. The filters were then individually wrapped in baked (500°C 5 hours) aluminum foil and frozen at -80°C prior to analysis in the lab, as described below.

Prior to sampling, the Nylon or polyester screens were cleaned using sequential rinses with HCl, DI, and MeOH, while GFF filters were combusted at 500°C for 5 hours.

In this study, 31 particle samples from below the OMZ core were collected across all 3 cruises. However, only 21 samples (7 small, 7 medium, 7 large) possessed the full suite of amino acids required for the mixing model and were used as separate sources in the model. For the purposes of the analysis, these samples are classified as "background samples."

During the Fall 2022 cruise, a small-scale test mining operation was conducted, generating a midwater mine waste plume for up to 32 hours of sample collection. Seven pumps were attached to a rosette equipped with a CTD (as described above), LISST, and transmissometer. Tow-Yo casts [42] were performed while the pumps were active, using real-time turbidity and beam transmission data from the transmissometer to locate the plume. While searching for the plume, we used <90% Beam Transmission as a guide to sampling plume particles. Coordination with the winch ensured the pumps remained within the plume as much as possible. Pumps were programmed to start 45 minutes after deployment to allow sufficient time to locate the plume and operated for 5 hours. These samples are referred to as "plume samples" in the text.

"Discharge samples" were collected aboard the test mining vessel *Hidden Gem* before the mining waste was discharged at depth. A similar size-fractionated filter system was used to separate particles into 3 size fractions. Three 142-mm diameter stainless steel filter holders were used sequentially to obtain the size fractionated particles. The filter holders were fitted with 53 μm or 6 μm Nylon or polyester screens or a 0.7 μm GFF filter. These samples were collected twice during the mining operation,

approximately 10 hours apart, for approximately 1 hour each time. The 53μm filters were replaced three times during each collection when this filter became clogged.

Supplementary Note 6: **LISST Particle Size Distribution**

In situ particle abundance and size distributions were characterized using a Laser In Situ Scatterometry and Transmissometry (LISST) sensor (LISST-DEEP Type-B, 650 nm, Sequoia Scientific) for 32 logarithmically spaced classes centered between 1.36 and 230.14 μm, with bandwidths ranging from 0.22 to 38 μm. The LISST was deployed *in situ* on the CTD rosette, both in background and midwater plume deployments, as well as concurrently with the McLane pumps. Data were processed using code customized from manufacturer supplied routines. First the scattering signal was corrected for background scattering due to pure water and any imperfections of the optics that would cause instrument drift (referred to as the "zscat" by the manufacturer). For this campaign we used the mean LISST raw scattering signal in the deepest 10 m of each cast (~ 1300-1400 m for plume casts). Raw counts (26.2 ± 5.8) from plume casts were slightly higher than our on-deck background measurements using de-ionized water from the ship's system (raw counts = 21.7 ± 10.5) however background spectra had similar shapes. While these backgrounds represent the lower detection limit of the LISST we do not assume them to be particle free; rather they represent particle minima for each cast. Background-corrected data were then inverted to obtain particle volume using the manufacturer provided spherical kernel which is calculated using Mie theory as a composite of several indices of refraction, and is designed to produce accurate inversion results over a broad range of particle types (ranging from organic to inorganic). After the inversion the data are corrected for the difference in laser power between the factory calibration and the *in situ* data, and an instrument-specific correction factor is applied to obtain the calibrated particle volume concentration, in units of volume particles per volume of water. These data are normalized to bin-width when plotting particle size distributions given log spacing of the size bins for the LISST. All data processing was

done in MATLAB using manufacturer-supplied code for the background scattering correction (getscat.m), the scattering inversion (invert.p using 15 iterations), and the laser power and concentration corrections (vdcorr.m).

Background particle concentrations were sampled in 3 casts, HC_050, HC_061, and MC_009. Plume particle concentrations were sampled in 6 casts, HC_053, HC_054, HC_056, HC_057, MC_004, and MC_006. Plume pump cast MC_004 had the highest concentration of particles observed among the 6 plume casts. This cast represents the most time spent within the plume (approximately 40% of the pump time) and thus most representative of within-plume conditions, and therefore used as the representative pump cast for LISST and dissolved oxygen analysis (below). The particle size distribution (PSD) of the midwater mining plume shows maxima in the 2.4-2.6 μm bin, suggesting dominance of very fine particles (<10 mm, Fig. S1). This matches both with the observed plume filters, as the 0.7-6 μm filter was heavily laden with material, as well as previous analyses of CCZ abyssal seafloor sedimentary particles ranging from <2 μm to 63 μm in size [43].

Table S1. **Total amino acid concentration in particles.** Mean and standard deviation of amino acid concentration normalized to particulate nitrogen (AA-ng / PN-µg) in Background and Mining-Derived material. Source data are provided in Source Data 1.

Pore	Background	Mining
0.7-6 µm	4.65 ± 2.49	3.84 ± 4.35
6-53 µm	41.08 ± 25.31	1.67 ± 1.46
>53 µm	46.25 ± 34.66	4.24 ± 4.67

Table S2. **Statistical analysis of background and mining particles.** Results of the one-way ANOVA tests comparing mean amino acid concentration between Background and Mining-Derived Material. *df*: degrees of freedom, *SS*: sums of squares, *MS*: mean squares, *F*: F value by permutation, *p*: *p*-value. Significant results shown in bold. Source data are provided in Source Data 1.

Pore	Variable	*df*	*SS*	*MS*	*F*	*p*
0.7-6 µm	Particle Type	1	1.875	1.875	0.200	0.663
0.7-6 µm	Residuals	12	112.454	9.371		
6-53 µm	Particle Type	1	3388.557	3388.557	6.795	**0.028**
6-53 µm	Residuals	9	4488.203	498.689		
>53 µm	Particle Type	1	5177.514	5177.514	5.573	**0.035**
>53 µm	Residuals	13	12077.781	929.060		

Table S3. **Maximum LISST particle concentration.** Maximum particle concentration (μL/L) of small (1.25-5.54 μm) and Large (5.54-250 μm) in 3 background and 6 plume casts from LISST. Source data are provided in Source Data 2.

Cast Type	Small	Large
Background	0.081	0.229
Plume	9.798	2.176

Table S4. **Maximum LISST particle count.** Maximum particle count (# x 10^8 /L) of small (1.25-5.54 μm) and Large (5.54-250 μm) in 3 background and 6 plume casts from LISST. Source data are provided in Source Data 6.

Cast Type	Small	Large
Background	0.135	0.003
Plume	11.154	0.117

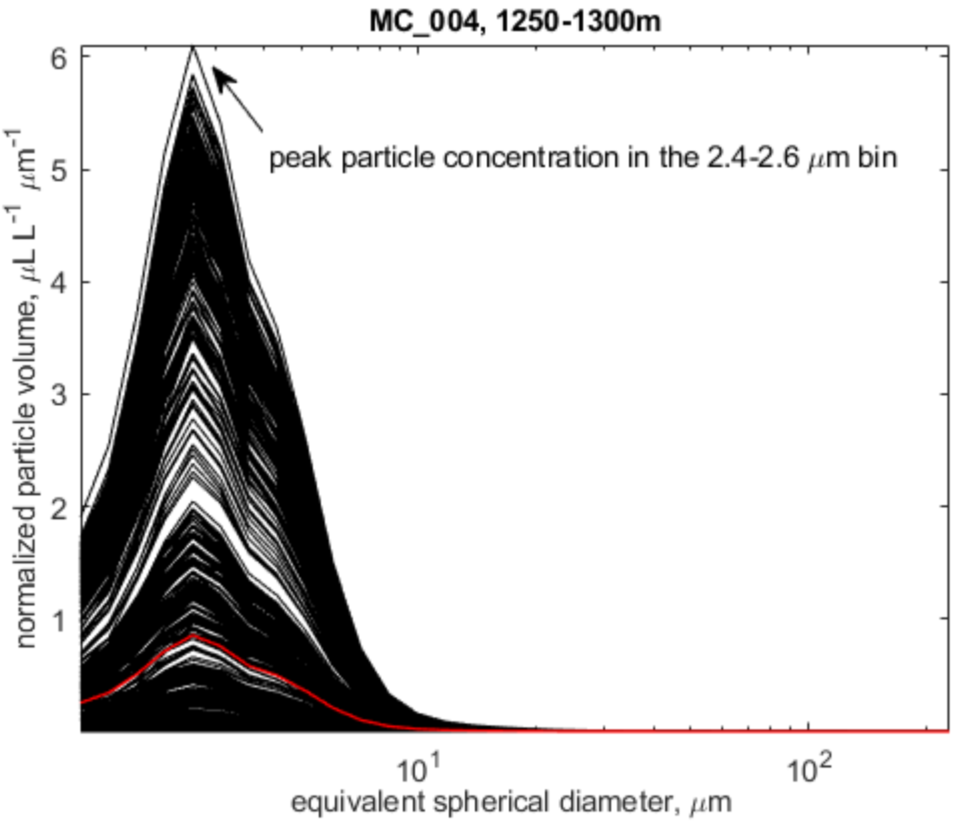

Fig. S1. LISST **size distribution of mining particles.** Plot of particle size distribution (semilog plot) of particle volume normalized to bin-width (ml L^{-1} mm^{-1}) at the depth of the midwater mining plume (1250-1300m) in plume pump cast (MC_004). The red line shows the average particle size distribution. Note the increase in abundance in particles < 10 µm. Source data are provided in Source Data 2.

Table S5. **Bayesian mixing model endmember values.** Mean and standard deviation of $\delta^{15}N_{Src,}$ $\delta^{13}C_{EAA}$ of background particles used in the Mixing Model. Source data are provided in Source Data 5.

Pore	Sample Size	$\delta^{15}N_{Phe}$	$\delta^{15}N_{Lys}$	$\delta^{13}C_{Leu}$
0.7-6 μm	7	10.64 ± 2.48	10.13 ± 3.36	-26.02 ± 1.26
6-53 μm	7	4.69 ± 2.59	2.15 ± 3.26	-28.35 ± 0.41
>53 μm	7	5.88 ± 2.41	6.25 ± 1.34	-27.82 ± 0.74

Table S6. **Statistical analysis of particle size fractions in mixing model.** Results of the two-sided pairwise PERMANOVA tests assessing the difference in $\delta^{15}N_{Src}/\delta^{13}C_{EAA}$ values across particle size fractions (small, medium, large). *df*: degrees of freedom, *SS*: sums of squares, *F*: F value by permutation, R^2: the effect size, *p*: *p*-value. p_{adj} : *p*-value adjusted (Bonferroni correction) based on 999 permutations. Significant results shown in bold. Source data are provided in Source Data 5.

Pairs	*df*	*SS*	*F*	*R2*	*p*	P_{adj}
0.7-6 µm vs >53 µm	1	143.361	10.535	0.467	0.004	**0.012**
0.7-6 µm vs 6-53 µm	1	366.240	20.042	0.625	0.001	**0.003**
>53 µm vs 6-53 µm	1	64.974	5.067	0.297	0.008	**0.024**

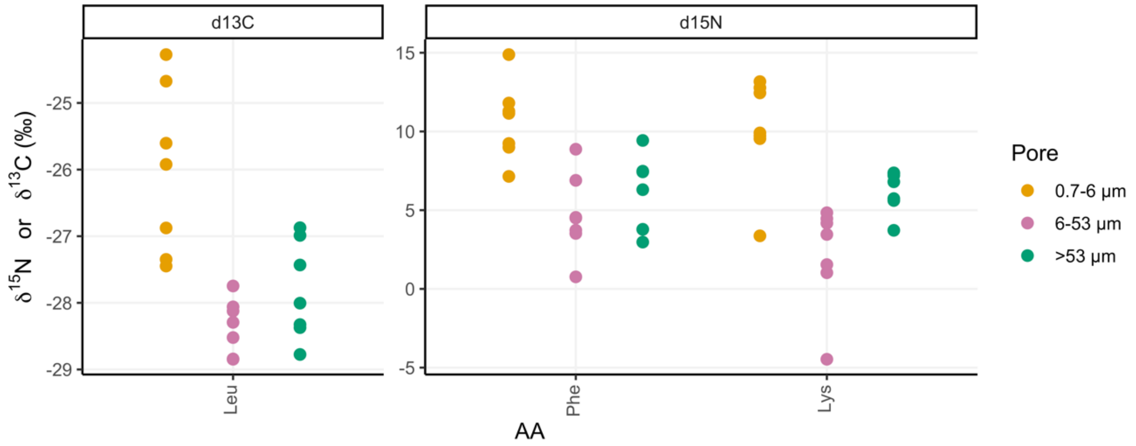

Fig. S2. **Bayesian mixing model endmember samples.** Plot of the $\delta^{15}N_{Src}$ and $\delta^{13}C_{EAA}$ in 0.7-6 μm, 6-53 μm and >53 μm size fractions of background particles used in the Mixing Model. Source data are provided in Source Data 5.

Table S7. **Averaged mixing model results of animal consumers.** Mean contribution of Small (0.7-6 μm) and Large (>6 μm) particles from Bayesian mixing model for consumers grouped by zooplankton size fraction/micronekton taxa. Standard deviation (SD) of combined results grouped by zooplankton size fraction/micronekton taxa. Source data are provided in Source Data 3.

Size Fraction/ Taxa	Small	Large	SD
0.2-0.5 mm	0.527	0.473	0.313
0.5-1.0 mm	0.313	0.687	0.281
1.0-2.0 mm	0.260	0.740	0.183
2.0-5.0 mm	0.343	0.657	0.247
Caridean	0.684	0.316	0.147
Cyclothone	0.411	0.589	0.322
Japatella	0.467	0.533	0.124
Mysid	0.536	0.464	0.168

Table S8. **Mining riser pipe oxygen concentration.** Summary of Winkler dissolved oxygen measurements from the riser pipe on the *Hidden Gem*.

Sample Type	Winkler O_2, µM
Riser Pipe	961.1
Riser Pipe	1061.1
Riser Pipe	921.5
Riser Pipe	770.1
Riser Pipe	956.5
Riser Pipe	914.4
Riser Pipe	1373.5

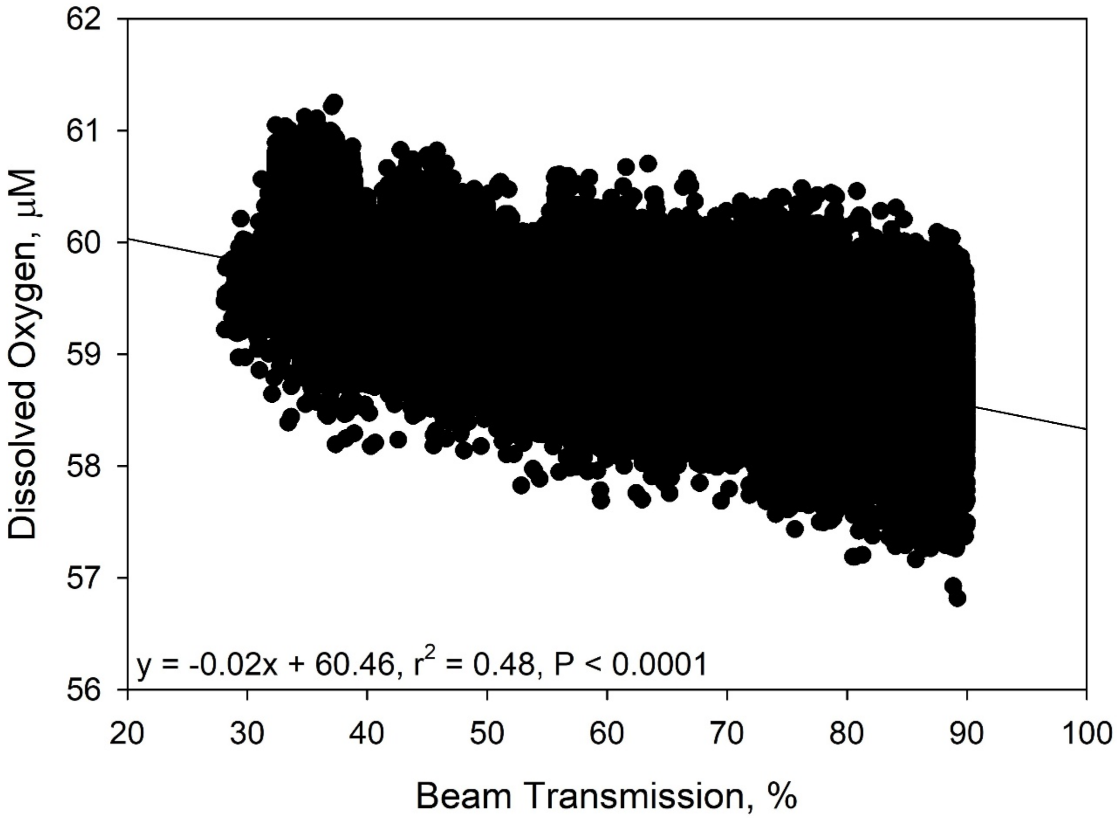

Figure S3. **CTD oxygen concentration inside a mining plume.** Plot of beam transmission versus dissolved oxygen concentration showing increased dissolved oxygen concentration with increasing particle contents in the mine waste plume. Beam transmission and dissolved oxygen concentrations were measured using sensors on the CTD rosette during a plume pump cast (MC_004) at the depth of the midwater mining plume (1253-1282m). Line in plot is least squares linear regression analysis (Type 1). Source data are provided in Source Data 7.

SI References

1. Wishner, K. F. *et al.* Ocean deoxygenation and zooplankton: Very small oxygen differences matter. *Sci Adv* **4**, eaau5180 (2018).
2. Ohkouchi, N. *et al.* Advances in the application of amino acid nitrogen isotopic analysis in ecological and biogeochemical studies. *Org Geochem* **113**, 150–174 (2017).
3. Chikaraishi, Y. *et al.* Determination of aquatic food-web structure based on compound-specific nitrogen isotopic composition of amino acids. *Limnol Oceanogr Methods* **7**, 740–750 (2009).
4. McClelland, J. W. & Montoya, J. P. Trophic relationships and the nitrogen isotopic composition of amino acids in plankton. *Ecology* **83**, 2173–2180 (2002).
5. McCarthy, M. D., Benner, R., Lee, C. & Fogel, M. L. Amino acid nitrogen isotopic fractionation patterns as indicators of heterotrophy in plankton, particulate, and dissolved organic matter. *Geochim Cosmochim Acta* **71**, 4727–4744 (2007).
6. Popp, B. N. *et al.* Insight into the Trophic Ecology of Yellowfin Tuna, Thunnus albacares, from Compound-Specific Nitrogen Isotope Analysis of Proteinaceous Amino Acids. *Terrestrial Ecology* **1**, 173–190 (2007).
7. Larsen, T. *et al.* Tracing Carbon Sources through Aquatic and Terrestrial Food Webs Using Amino Acid Stable Isotope Fingerprinting. *PLoS One* **8**, e73441 (2013).
8. Fantle, M. S., Dittel, A. I., Schwalm, S. M., Epifanio, C. E. & Fogel, M. L. A food web analysis of the juvenile blue crab, *Callinectes sapidus*, using stable isotopes in whole animals and individual amino acids. *Oecologia* **120**, 416–426 (1999).
9. Larsen, T., Taylor, D. L., Leigh, M. B. & O'Brien, D. M. Stable isotope fingerprinting: a novel method for identifying plant, fungal, or bacterial origins of amino acids. *Ecology* **90**, 3526–3535 (2009).

10. Larsen, T. *et al.* Tracing carbon sources through aquatic and terrestrial food webs using amino acid stable isotope fingerprinting. *PLoS One* **8**, e73441 (2013).

11. Sinniger, F. *et al.* Worldwide analysis of sedimentary DNA reveals major gaps in taxonomic knowledge of deep-sea benthos. *Front Mar Sci* **3**, (2016).

12. Fonseca, V. G. *et al.* Second-generation environmental sequencing unmasks marine metazoan biodiversity. *Nat Commun* **1**, 98 (2010).

13. Bolyen, E. *et al.* Reproducible, interactive, scalable and extensible microbiome data science using QIIME 2. *Nat Biotechnol* **37**, 852–857 (2019).

14. Callahan, B. J. *et al.* DADA2: High-resolution sample inference from Illumina amplicon data. *Nat Methods* **13**, 581–583 (2016).

15. Bokulich, N. A. *et al.* Optimizing taxonomic classification of marker-gene amplicon sequences with QIIME 2's q2-feature-classifier plugin. *Microbiome* **6**, 90 (2018).

16. Guillou, L. *et al.* The Protist Ribosomal Reference database (PR2): A catalog of unicellular eukaryote Small Sub-Unit rRNA sequences with curated taxonomy. *Nucleic Acids Res* **41**, D597-604 (2013).

17. Quast, C. *et al.* The SILVA ribosomal RNA gene database project: Improved data processing and web-based tools. *Nucleic Acids Res* **41**, D590-6 (2013).

18. O'Brien, T. D., Blanco-Bercial, L., Questel, J. M., Batta-Lona, P. G. & Bucklin, A. MetaZooGene Atlas and Database: Reference Sequences for Marine Ecosystems. in *DNA Barcoding: Methods and Protocols* (ed. DeSalle, R.) 475–489 (Springer US, New York, NY, 2024). doi:10.1007/978-1-0716-3581-0_28.

19. Bucklin, A. *et al.* Toward a global reference database of COI barcodes for marine zooplankton. *Mar Biol* **168**, 78 (2021).

20. Benson, D. A. *et al.* GenBank. *Nucleic Acids Res* **41**, (2013).

21. Davis, N. M., Proctor, Di. M., Holmes, S. P., Relman, D. A. & Callahan, B. J. Simple statistical identification and removal of contaminant sequences in marker-gene and metagenomics data. *Microbiome* **6**, 226 (2018).
22. Benedetti, F., Gasparini, S. & Ayata, S. D. Identifying copepod functional groups from species functional traits. *J Plankton Res* **38**, 159–166 (2016).
23. Homma, T., Yamaguchi, A., Bower, J. R. & Imai, I. Vertical changes in abundance, biomass, and community structure of copepods in the northern North Pacific and Bering Sea at 0-3,000 m depth, and their role on the vertical flux of surface-produced organic material. *Bulletin of the Faculty of Fisheries Sciences, Hokkaido University* **61**, 29–47 (2011).
24. Sano, M., Maki, K., Nishibe, Y., Nagata, T. & Nishida, S. Feeding habits of mesopelagic copepods in Sagami Bay: Insights from integrative analysis. *Prog Oceanogr* **110**, 11–26 (2013).
25. Nishida, S. & Ohtsuka, S. Ultrastructure of the mouthpart sensory setae in mesopelagic copepods of the family Scolecitrichidae. *Plankton Biol. Ecol.* **1/2**, 81–90 (1997).
26. Matthews, S. A., Goetze, E., Ohman, M. D. & Hauser, L. Recommendations for interpreting zooplankton metabarcoding and integrating molecular methods with morphological analyses. *ICES Journal of Marine Science* **78**, 3387–3396 (2021).
27. Ershova, E. A. *et al.* Metabarcoding as a quantitative tool for estimating biodiversity and relative biomass of marine zooplankton. *ICES Journal of Marine Science* **78**, 3342–3355 (2021).
28. Torres, J. J. & Childress, J. J. Respiration and chemical composition of the bathypelagic euphausiid *Bentheuphausia amblyops*. *Mar Biol* **87**, 267–272 (1985).
29. Flock, M. E. & Hopkins, T. L. Species Composition, Vertical Distribution, and Food Habits of the Sergestid Shrimp Assemblage in the Eastern Gulf of Mexico. *Journal of Crustacean Biology* **12**, 210–223 (1992).

30. Mincks, S. L. *et al.* Distribution, abundance, and feeding ecology of decapods in the Arabian Sea, with implications for vertical flux. *Deep-Sea Research II* **47**, 1475–1516 (2000).

31. Hopkins, T. L., Flock, M. E., Gartner, J. V. J. & Torres, J. J. Structure and trophic ecology of a low latitude midwater decapod and mysid assemblage. *Mar Ecol Prog Ser* 109, 143–156 (1994).

32. Drazen, J. C. & Sutton, T. T. Dining in the Deep: The Feeding Ecology of Deep-Sea Fishes. *Ann Rev Mar Sci* **9**, 337–366 (2017).

33. Gartner Jr., J. V & Musick, J. A. Feeding habits of the deep-sea fish, *Scopelogadus beanii* (Pisces: Melamphaidae), in the western North Atlantic. *Deep Sea Research* **36**, 1457–1469 (1989).

34. Ebeling, A. W. & Cailliet, G. M. Mouth size and predator strategy of midwater fishes. *Deep Sea Research* **21**, 959–968 (1974).

35. Bartow, K. A. & Sutton, T. T. Taxonomy and Ecology of the Deep-pelagic Fish Family Melamphaidae with Emphasis on Interactions with a Mid-Ocean Ridge System. *Charles E. Schmidt College of Science* vol. Ph.D. (Boca Raton, Fl, 2010).

36. SeaLifeBase. www.sealifebase.org (2024).

37. Heffernan, J. J. & Hopkins, T. L. Vertical Distribution and Feeding of the Shrimp Genera Gennadas and Bentheogennema (Decapoda: Penaeidea) in the Eastern Gulf of Mexico. *Journal of Crustacean Biology* **1**, 461–473 (1981).

38. Zhou, F., Hirai, J., Hamasaki, K., Horii, S. & Tsuda, A. Feeding Ecology of Three Euphausiid Species in the North Pacific Ocean Inferred From 18S V9 Metabarcoding and Stable Isotope Analysis. *Front Mar Sci* **8**, (2021).

39. Ariza, A., Garijo, J. C., Landeira, J. M., Bordes, F. & Hernández-León, S. Migrant biomass and respiratory carbon flux by zooplankton and micronekton in the subtropical northeast Atlantic Ocean (Canary Islands). *Prog Oceanogr* **134**, 330–342 (2015).

40. Burukovsky, R. N. & Falkenhaug, T. Feeding of the pelagic shrimp *Acanthephyra pelagica* (Risso, 1816) (Crustacea: Decapoda: Oplophoridae) in the northern Mid-Atlantic Ridge area in 1984 and 2004. *Arthropoda Sel* **24**, 303–316 (2015).

41. Bishop, J. K. B., Lam, P. J. & Wood, T. J. Getting good particles: Accurate sampling of particles by large volume in-situ filtration. *Limnol Oceanogr Methods* **10**, 681–710 (2012).

42. Baker, E. T., Lavelle, J. W. & Massoth, G. J. Hydrothermal particle plumes over the southern Juan de Fuca Ridge. *Nature* **316**, 342–344 (1985).

43. Zawadzki, D., Maciag, Ł., Abramowski, T. & McCartney, K. Fractionation Trends and Variability of Rare Earth Elements and Selected Critical Metals in Pelagic Sediment from Abyssal Basin of NE Pacific (Clarion-Clipperton Fracture Zone). *Minerals* **10**, 1–38 (2020).
