# Supplementary material for: Deep-sea mining discharge can disrupt midwater food webs
Source: Nat Commun. 2025 Nov 6;16:9575. doi: 10.1038/s41467-025-65411-w (PMC12592452; doi:10.1038/s41467-025-65411-w)
Supplement: Supplementary file 2 — Descriptions of Additional Supplementary Files [file 41467_2025_65411_MOESM2_ESM.pdf]

Description of Additional Supplementary Files

Supplementary Data 1: Zooplankton functional groups. Zooplankton feeding ecology for core taxa sampled between 700 – 1500m (186 ASVs). Taxon is derived from metabarcoding read classification, with feeding type and particle dependence categorized based on literature reports. Core taxa include ASVs present in >0.1% of total relative read abundance within a given tow and present in at least two MOCNESS tows. Taxa with names highlighted in bold were present at the potential discharge depth between 1000 - 1500m. ◊ indicates taxa with classification that may be to a close relative, due to the limitations of reference databases (missing taxa).

Supplementary Data 2: Micronekton functional groups. Taxa found from 700 - 1500 m and their functional group classification. Those listed with a CD (e.g. 'Cannot Determine') mean that the LTU is too broad to determine, or no data are available. Highlighted rows are specific taxa sampled for CSIA-AA mixing model diet analysis. Taxa were selected as they were abundant representatives of functional groups collected in both the lower oxycline and suboxycline.

Supplementary Data 3: Zooplankton sequencing data. Metabarcoding of bulk pelagic zooplankton.

Supplementary Data 4: Bayesian consumer data. Amino acid isotope values for animal consumers used in Bayesian micing model.

Supplementary Code 1: Bayesian mixing model code. Model code, instructions to install and run code, data inputs, and expected data outputs.
