# Supplementary material for: Deep-sea mining discharge can disrupt midwater food webs
Source: Nat Commun. 2025 Nov 6;16:9575. doi: 10.1038/s41467-025-65411-w (PMC12592452; doi:10.1038/s41467-025-65411-w)
Supplement: Supplementary file 4 — Supplementary Code 1 [file 41467_2025_65411_MOESM4_ESM.zip › Supplementary Code 1/Readme.docx]

This folder contains demo code and data for the Bayesian Mixing model used in Dowd M.H., Assad V.E., Cazares-Nuesser A.E., Drazen J.C., Goetze E., White A.E., and Popp B.N. (2025) Disruptions to Midwater Food Webs from Deep-Sea Mining Discharge. Nature Communications.

To run this code, JAGS needs to be downloaded and installed from <https://sourceforge.net/projects/mcmc-jags/files/>. Follow to this link, download the latest version, and run the downloaded file. After that, necessary packages in R can be installed using the install.packages(…) command as normal.

The mixing models are R Markdown files. It was created and tested in R version 4.2.2 (2022-10-31) on macOS Ventura version 13.0.1, R version 4.4.0 on macOS 15.5, and R version 4.4.1 on Windows 11 Pro. Packages required are in the first chunk, which can be installed via install.packages(). Installation of packages should take less than 10 minutes if none have been installed.

As long as the packages in the first setup chunk are installed, you should be able to click “Knit” and it will run without any changes necessary. If it has an error in the setup chunk involving the “setwd(dirname(rstudioapi::getActiveDocumentContext()$path))” line, running the chunk by itself with the green play button, or highlighting and running everything within the chunk, and then pressing “Knit” should fix the problem.

I have set up this demo code so that it is self contained within the folder. You should not need to change the file destination as long as the demo remains within the folder. If you run into issues where it cannot find the file directory, use the search function and type in “Find me” to find where you may need to change the directory link.

There are two demos of the Bayesian mixing model in the folder, one for Japatella octopods, and one for Zooplankton 1.0-2.0 mm in size from the Fall 2021 cruise. In the paper, each set of samples were run 100,000 times on 3 chains. Issues with memory necessitated splitting the consumers into individual runs by taxa/size/season.

This demo only runs 5,000 times on 3 chains (samsPerChain). It takes approximately 30 seconds to run (as opposed to 20-40 minutes for the full model).

When the demo is knit, it will create 2 folder, ending with _cache and _files, as well as producing an .html output. You can view the .html to see the Particle Source as in Fig S2. Diagnostics are also included to make sure the chains are behaving properly. Further diagnostics were removed from the demo for ease of running and reduction in time.

The final chunk will create an excel output of the mean contribution of particle sizes per sample, as in Table S7. This will be sent to the Excel_Sheets folder.

Already in the Excel_Sheets folder are the Background Particle Source Data (Data_Sources), the Consumer Data (Data_Japatella, Data_10_Fall; already split so excess filtering of data is not needed in code), and Mean Contribution data (Mean_Contribution_Japatella, Mean_Contribution_10_Fall; Table S7 already split per consumer type). In the Dowd_Demo folder are the expected outputs of running the 2 demo mixing models.

When you Knit the demos, it will create sheets that should be identical to those in the Dowd_Demo folder. You can also compare the output of the demo to that of data from the paper; you will see the values are nearly identical, differences due to the reduced number of samples run per chain in the demo.

The _cache, _files, and .html outputs can all be deleted after knitting, all together or in any combination. Re-Knitting the .rmd will recreate the folders, output, and reproduce the excel output.
