# Supplementary material for: Deep-sea mining discharge can disrupt midwater food webs
Source: Nat Commun. 2025 Nov 6;16:9575. doi: 10.1038/s41467-025-65411-w (PMC12592452; doi:10.1038/s41467-025-65411-w)
Supplement: Supplementary file 5 — Reporting Summary [file 41467_2025_65411_MOESM5_ESM.pdf]

nature portfolio

Corresponding author(s): Michael Dowd

Last updated by author(s): Aug 28, 2025

Reporting Summary

Nature Portfolio wishes to improve the reproducibility of the work that we publish. This form provides structure for consistency and transparency in reporting. For further information on Nature Portfolio policies, see our Editorial Policies and the Editorial Policy Checklist.

Statistics

For all statistical analyses, confirm that the following items are present in the figure legend, table legend, main text, or Methods section.

n/a	Confirmed	
☐	☒	The exact sample size (*n*) for each experimental group/condition, given as a discrete number and unit of measurement
☐	☒	A statement on whether measurements were taken from distinct samples or whether the same sample was measured repeatedly
☐	☒	The statistical test(s) used AND whether they are one- or two-sided<br>*Only common tests should be described solely by name; describe more complex techniques in the Methods section.*
☐	☒	A description of all covariates tested
☒	☐	A description of any assumptions or corrections, such as tests of normality and adjustment for multiple comparisons
☐	☒	A full description of the statistical parameters including central tendency (e.g. means) or other basic estimates (e.g. regression coefficient) AND variation (e.g. standard deviation) or associated estimates of uncertainty (e.g. confidence intervals)
☐	☒	For null hypothesis testing, the test statistic (e.g. *F*, *t*, *r*) with confidence intervals, effect sizes, degrees of freedom and *P* value noted<br>*Give P values as exact values whenever suitable.*
☐	☒	For Bayesian analysis, information on the choice of priors and Markov chain Monte Carlo settings
☒	☐	For hierarchical and complex designs, identification of the appropriate level for tests and full reporting of outcomes
☒	☐	Estimates of effect sizes (e.g. Cohen's *d*, Pearson's *r*), indicating how they were calculated

Our web collection on statistics for biologists contains articles on many of the points above.

Software and code

Policy information about availability of computer code

Data collection	No software was used for data collection
Data analysis	Data was analyzed using open-source code in R (Version 4.2.2) using the packages runjags (version 2.2.2-4), dirichletReg (0.7-1), MASS (version 7.3-58.1), pairwiseAdonis (version 0.4.1), vegan (version 2.6-4) 57, and gglopt2 (version 3.5.1) 58. Model Code is provided with this paper. All model code and dependencies are publicly available in a GitHub repository (https://github.com/mdowd3/Mining-FoodWeb-CSIA).

For manuscripts utilizing custom algorithms or software that are central to the research but not yet described in published literature, software must be made available to editors and reviewers. We strongly encourage code deposition in a community repository (e.g. GitHub). See the Nature Portfolio guidelines for submitting code & software for further information.

Data

Policy information about availability of data

All manuscripts must include a data availability statement. This statement should provide the following information, where applicable:

- Accession codes, unique identifiers, or web links for publicly available datasets
- A description of any restrictions on data availability
- For clinical datasets or third party data, please ensure that the statement adheres to our policy

All isotopic, Bayesian mixing model, and LISST data generated in this study are publicly available via GitHub and linked to Zenodo (https://github.com/mdowd3/Mining-FoodWeb-CSIA). All data are also available in the International Seabed Authority DeepData Database (https://www.isa.org.jm/deepdata-database/). Zooplankton sequence data are available in the NCBI Sequence Read Archive under BioProject PRJNA1254332 and SRA accession numbers SRR33375556 to

SRR33375942 (https://www.ncbi.nlm.nih.gov/bioproject/PRJNA1254332). Isotopic, Bayesian mixing model, and LISST source data generated in this study are provided in Source Data 1-7. Zooplankton and micronekton functional ecology data generated in this study are provided in Supplementary Data 1-2. Zooplankton sequence data generated in this study are provided in Supplementary Data 3. Bayesian consumer data generated in this study are provided in Supplementary Data 4.

Research involving human participants, their data, or biological material

Policy information about studies with human participants or human data. See also policy information about sex, gender (identity/presentation), and sexual orientation and race, ethnicity and racism.

Reporting on sex and gender	*Use the terms sex (biological attribute) and gender (shaped by social and cultural circumstances) carefully in order to avoid confusing both terms. Indicate if findings apply to only one sex or gender; describe whether sex and gender were considered in study design; whether sex and/or gender was determined based on self-reporting or assigned and methods used.*<br>*Provide in the source data disaggregated sex and gender data, where this information has been collected, and if consent has been obtained for sharing of individual-level data; provide overall numbers in this Reporting Summary. Please state if this information has not been collected.*<br>*Report sex- and gender-based analyses where performed, justify reasons for lack of sex- and gender-based analysis.*
Reporting on race, ethnicity, or other socially relevant groupings	*Please specify the socially constructed or socially relevant categorization variable(s) used in your manuscript and explain why they were used. Please note that such variables should not be used as proxies for other socially constructed/relevant variables (for example, race or ethnicity should not be used as a proxy for socioeconomic status).*<br>*Provide clear definitions of the relevant terms used, how they were provided (by the participants/respondents, the researchers, or third parties), and the method(s) used to classify people into the different categories (e.g. self-report, census or administrative data, social media data, etc.)*<br>*Please provide details about how you controlled for confounding variables in your analyses.*
Population characteristics	*Describe the covariate-relevant population characteristics of the human research participants (e.g. age, genotypic information, past and current diagnosis and treatment categories). If you filled out the behavioural & social sciences study design questions and have nothing to add here, write "See above."*
Recruitment	*Describe how participants were recruited. Outline any potential self-selection bias or other biases that may be present and how these are likely to impact results.*
Ethics oversight	*Identify the organization(s) that approved the study protocol.*

Note that full information on the approval of the study protocol must also be provided in the manuscript.

Field-specific reporting

Please select the one below that is the best fit for your research. If you are not sure, read the appropriate sections before making your selection.

☐ Life sciences ☐ Behavioural & social sciences ☒ Ecological, evolutionary & environmental sciences

For a reference copy of the document with all sections, see nature.com/documents/nr-reporting-summary-flat.pdf

Ecological, evolutionary & environmental sciences study design

All studies must disclose on these points even when the disclosure is negative.

Study description	This study employs an open source Bayesian mixing model to estimate the proportion of size-fractionated particles that contribute to the base of a mesopelagic faunal food web. The model incorporates particles from 3 size fractions (n=7 each size fraction) as the potential base of the food web, and analyzes 45 animals, including 24 zooplankton and 21 micronekton.
Research sample	Bulk zooplankton were collected and sieved into size fractions, while micronekton were collected and sorted by species. Specific micronekton taxa analyzed included Acanthephyra brevicarinata (caridean shrimp), Cyclothone spp. (fish), Japatella spp. (octopod), and Eucopia spp. (mysid shrimp). The isotopic results of these size fractionated zooplankton and specific micronekton taxa were used in the mixing model. These organisms represent lower trophic position organisms that rely on particles as the base of the food web.
Sampling strategy	Particle samples were collected over the course of 3 cruises conducted in different seasons and years. We found no difference in isotope values between cruises and sites, allowing samples to be pooled and used as endmembers in the mixing model.<br>Bulk zooplankton samples were collected in Spring and Fall 2021 and were size fractionated for every net within every tow. Each size fractionated zooplankton sample analyzed in this study represents a different net/tow. Isotope results showed no difference within a size fraction across cruises and depths, permitting their use as consumers in the mixing model.<br>Micronekton samples were collected in Fall 2021 and were sorted for each net within every tow. Each micronekton sample analyzed in this study represents a different individual specimen. The results of isotope analysis revealed no difference within a taxa across depths, allowing these results to be used as consumers in the mixing model.<br>Sample sizes were determined based on the availability of particle pumps casts, zooplankton net tows, and identifiable micronekton samples.<br>Plume and discharge samples were collected only twice due to the short duration of plume generation.
Data collection	Particle samples were collected using an in situ pump system, led by Brian Popp and Michael Dowd. Zooplankton were collected with

	1m2 MOCNESS nets and size fractionated using mesh sieves. Sampling and processing at sea were led by Erica Goetze and Alexus Cazares-Nuesser. Micronekton were collected with 10m2 MOCNESS nets, sorted into groups by Family at sea, and later identified to the lowest taxonomic unit based by morphology. Micronekton collection and analysis were led by Jeff Drazen and Victoria Assad. Isotopic analyses of particles, zooplankton, and micronekton were conducted by Michael Dowd.
Timing and spatial scale	Samples were collected in the NORI-D mining claim in the Clarion-Clipperton Zone (CCZ) during 3 cruises: DG5B in Spring (March-April) 2021, DG5C in Fall (October) 2021, and DG7B in Fall (October-November) in 2022. Particle samples were collected during all 3 cruises, while zooplankton were collected in the Spring and Fall 2021 cruises. Micronekton samples analyzed were only collected in the Fall 2021 cruise. Net tows and particle pump casts were conducted throughout these sampling periods. Net tows were performed at night and during the day to account for active diel vertical migration, whereas particle samples were conducted whenever possible to accommodate the schedule, as these samples are not influenced by time of day. Samples were collected at two sites, approximately 60 nm apart.<br>Plume and discharge samples were collected during the Fall 2022 cruise on 10/25/22-10/26/22. Samples occurred at the beginning and end of the discharge process to obtain multiple samples and facilitate the collection efforts of other scientists.
Data exclusions	Particle and animal samples that did not have the necessary amino acid stable isotope data for the mixing model were excluded.
Reproducibility	Isotopic samples were analyzed in triplicate whenever possible, with data reported as the mean ± standard deviation. Standard background correction was performed to ensure reproducibility of results.
Randomization	We found no difference in the isotope values between cruises or sites, so samples covariates did not need to be randomized.
Blinding	Data was not blinded as it is not necessary in a Bayesian mixing model.

Did the study involve field work? [x] Yes [ ] No

Field work, collection and transport

Field conditions	Samples were collected over 3 cruises, each lasting approximately one month. During the first Spring 2021 cruise (late March-April), an anti-cyclonic eddy passed through the sampling area. Aside from persistent differences in upper-ocean oxygen structure between the Spring and Fall cruises, no other relevant environmental variations were observed.
Location	Samples were collected in the Clarion Clipperton Zone, at the NORI-D sample collection site, approximately 10 N, 117 W, water depth approximately 4300m.
Access & import/export	All cruises mobilized and de-mobilized at US ports, so no import-export permits were required. All field work was in international waters.
Disturbance	Vessel employed procedures for collection and combustion of trash as standard to at-sea science research vessels to minimize dumping of trash and disturbance to the ecosystem. Waste disposal at sea followed the internationally recognized standards included in the MARPOL Annex IV and V regulations.<br>All possible efforts were taken to minimize disturbance to the environment.

Reporting for specific materials, systems and methods

We require information from authors about some types of materials, experimental systems and methods used in many studies. Here, indicate whether each material, system or method listed is relevant to your study. If you are not sure if a list item applies to your research, read the appropriate section before selecting a response.

Materials & experimental systems

n/a	Involved in the study	
x		Antibodies
x		Eukaryotic cell lines
x		Palaeontology and archaeology
	x	Animals and other organisms
x		Clinical data
x		Dual use research of concern
x		Plants

Methods

n/a	Involved in the study	
x		ChIP-seq
x		Flow cytometry
x		MRI-based neuroimaging

Animals and other research organisms

Policy information about studies involving animals; ARRIVE guidelines recommended for reporting animal research, and Sex and Gender in Research

Laboratory animals	No laboratory animals were used in this study.

Wild animals	All animal handling and sampling were state of the art and are in accordance with the American Society of Ichthyologists and Herpotologists “Guidelines for Use of Fishes in Field Research.” To minimize pain and stress in the captured animals, they were euthanized ASAP after capture if they were still alive. Any that were alive but not needed returned to the ocean as recommended by the ASIH guidelines. The smallest number of animals were used to achieve statistical validity of isotopic analyses for food web analysis. All fishes were collected under UH IACUC permit 14-1934-9.
Reporting on sex	No consideration of the sex of wild animals was made.
Field-collected samples	No laboratory work was conducted on wild animals.
Ethics oversight	UH IACUC approved this study protocol.

Note that full information on the approval of the study protocol must also be provided in the manuscript.

Plants

Seed stocks	*Report on the source of all seed stocks or other plant material used. If applicable, state the seed stock centre and catalogue number. If plant specimens were collected from the field, describe the collection location, date and sampling procedures.*
Novel plant genotypes	*Describe the methods by which all novel plant genotypes were produced. This includes those generated by transgenic approaches, gene editing, chemical/radiation-based mutagenesis and hybridization. For transgenic lines, describe the transformation method, the number of independent lines analyzed and the generation upon which experiments were performed. For gene-edited lines, describe the editor used, the endogenous sequence targeted for editing, the targeting guide RNA sequence (if applicable) and how the editor was applied.*
Authentication	*Describe any authentication procedures for each seed stock used or novel genotype generated. Describe any experiments used to assess the effect of a mutation and, where applicable, how potential secondary effects (e.g. second site T-DNA insertions, mosiacism, off-target gene editing) were examined.*
